# A Cell Culture Chip with Transparent, Micropillar-Decorated Bottom for Live Cell Imaging and Screening of Breast Cancer Cells

**DOI:** 10.3390/mi13010093

**Published:** 2022-01-07

**Authors:** Menekse Ermis, Ezgi Antmen, Ozgur Kuren, Utkan Demirci, Vasif Hasirci

**Affiliations:** 1 BIOMATEN, Center of Excellence in Biomaterials and Tissue Engineering, Middle East Technical University, Ankara 06800, Turkey; e.menek@gmail.com (M.E.); ezgiantmenn@gmail.com (E.A.); ozgur.kuren97@gmail.com (O.K.); 2 Canary Center for Cancer Early Detection, Department of Radiology, Electrical Engineering Department, Stanford University, Palo Alto, CA 94305, USA; utkan@stanford.edu; 3 Department of Medical Engineering, Acibadem Mehmet Ali Aydinlar University, Istanbul 34684, Turkey; 4 ACU Biomaterials Center, Acibadem Mehmet Ali Aydinlar University, Istanbul 34684, Turkey

**Keywords:** micropatterns, breast cancer, imaging chip, proliferation, apoptosis

## Abstract

In the recent years, microfabrication technologies have been widely used in cell biology, tissue engineering, and regenerative medicine studies. Today, the implementation of microfabricated devices in cancer research is frequent and advantageous because it enables the study of cancer cells in controlled microenvironments provided by the microchips. Breast cancer is one of the most common cancers in women, and the way breast cancer cells interact with their physical microenvironment is still under investigation. In this study, we developed a transparent cell culture chip (Ch-Pattern) with a micropillar-decorated bottom that makes live imaging and monitoring of the metabolic, proliferative, apoptotic, and morphological behavior of breast cancer cells possible. The reason for the use of micropatterned surfaces is because cancer cells deform and lose their shape and acto-myosin integrity on micropatterned substrates, and this allows the quantification of the changes in morphology and through that identification of the cancerous cells. In the last decade, cancer cells were studied on micropatterned substrates of varying sizes and with a variety of biomaterials. These studies were conducted using conventional cell culture plates carrying patterned films. In the present study, cell culture protocols were conducted in the clear-bottom micropatterned chip. This approach adds significantly to the current knowledge and applications by enabling low-volume and high-throughput processing of the cell behavior, especially the cell–micropattern interactions. In this study, two different breast cancer cell lines, MDA-MB-231 and MCF-7, were used. MDA-MB-231 cells are invasive and metastatic, while MCF-7 cells are not metastatic. The nuclei of these two cell types deformed to distinctly different levels on the micropatterns, had different metabolic and proliferation rates, and their cell cycles were affected. The Ch-Pattern chips developed in this study proved to have significant advantages when used in the biological analysis of live cells and highly beneficial in the study of screening breast cancer cell–substrate interactions in vitro.

## 1. Introduction

Microfabrication technologies provide a platform to study cell–substrate interactions systematically [1,2,3]. Today, microfabricated devices for cell studies have a range of applications from studying cell biology [4] to detecting disease [5,6,7,8,9], differentiating cells [5,7,8,9,10,11,12], producing tissue models [10,11,12,13,14], and modifying implant surfaces for enhanced tissue integration [15,16,17,18,19,20]. Lab-on-a-chip devices allow the integration of cell capture, detection, drug delivery, and tissue engineering applications on a single device and can also use microfabrication technologies while doing so [21,22,23,24,25,26]. One of the standard methods for studying cell–substrate and cell–cell interactions is microscopy [27,28,29,30,31,32]. Today, light microscopes allow a sophisticated analysis of cells and subcellular compartments by the help of the fluorescence principle. A fluorescence probe is excited under a light source with a certain wavelength (and energy) and emits light in response. This allows the imaging of subcellular structures tagged with desired fluorescence probes with a dark background and increases resolution and pattern recognition by the human eye and computers. Using fluorescence microscopy and associated techniques (widefield fluorescence, confocal laser scanning microscope, spinning disk confocal, multiphoton confocal, live-cell imaging, etc.), cells and cellular processes can be visualized under static or dynamic cell culture conditions [33,34,35,36]. Microscopy techniques are being developed for the imaging of complex samples (in addition to the conventional histology sections), and application-specific devices are in need. Microfabricated devices can meet the need for these devices to be used in live cell imaging and analysis. Transparent devices allow the undisturbed microscopic imaging of cells in their microenvironment and observation of changes or interventions in real time.

Cancer is a disease in which cells proliferate uncontrollably without responding to signals restricting cell division and form tissues that do not show the same organization as healthy tissues. They divide more rapidly and are immortal. In the last decade, studies on the relation between the biophysical properties of cells and cancer progression have significantly increased [37]. The mechanical properties of cancer cells and their interaction with the microenvironment are required to understand the nature of the cancer. Forces generated in the imminent microenvironment of a cancer cell are sensed and conveyed intracellularly. This is called mechanosensing. These forces are then transduced to the cell interior through ‘mechanotransduction’. These mechanosensing and mechanotransduction pathways are very active in cancer cells. The transnuclear mechanism that transmits cytoplasmic force signals to the nucleus is called the Linker of Nucleoskeleton and Cytoskeleton (LINC) complex. The LINC complex proteins and other mechanotransduction molecular components are regulated in cancer cells and cells from healthy tissues [37,38,39]. Eventually, external force signals that reach the nuclei can affect chromatin condensation, gene and protein expressions, and various cellular activities such as differentiation, proliferation, and apoptosis. These signals evoke different responses in cancer and healthy cells. They promote proliferation and uncontrolled growth in the cancer cells and differentiation in the healthy ones. It is known that the nuclei of cancer cells are softer than those of healthy cells. On micropatterned surfaces, these nuclei show different levels of morphological distortions as a result of force transduction, which helps migration and metastasis [40,41,42,43,44]. Therefore, the deformability of cancer cells, especially their nuclei, is being studied in the discrimination of healthy and diseased cells.

There are several methods for the quantification of physical properties of cancer cells, and the most common conventional techniques in cell mechanics are atomic force microscopy (AFM), optical tweezers, micropipettes, and confocal and fluorescence microscopy [38]. All these methods have advantages and disadvantages, but most are laborious and take time, and their use for high-throughput analysis is limited. Biomaterials gained the spotlight several decades ago with the increased need for tissue and organ transplantation and the limited donor tissue supply [39]. Since its foundation, numerous biomaterials were developed for tissue engineering. Today, almost all organs and tissues are being modeled and mimicked in vitro with different degrees of success. Biomaterials also allowed us to study cells by creating microenvironments to study in vitro cell–material interactions in these controlled environments that can mimic healthy and diseased conditions with more controlled variables than traditional 2D cultures [40]. Biomaterials can be used to microfabricate surfaces. Both surface chemical and physical properties can be controlled, and microfabrication allows the precise control of surface topography [41]. Using such microfabricated substrates made from biomaterials enables us to study cancer cells and their responses to their physical microenvironment [42]. These micropatterned substrates give us the opportunity to study the morphology, differentiation, and metabolic changes in cancer cells with this refined but relatively simple (from the production and characterization point of view) tool.

The use of biomaterials is a must because they are introduced to the human body and interact with the biomolecules, cells, and tissues. Studies conducted using a material not compatible with the biological environment would not be suitable to derive conclusions applicable to the human body.

Micropatterned surfaces have been used in cell biology for the last two decades. Dike et al. showed that confining endothelial cells into microchannels with 10 µm width lines induced the morphology of the cells toward forming vascular tubes. They also showed that larger channels with 30 µm width did not induce the same morphological change and elongation, and only proliferation was observed [43]. In another study, microdomains with 100 µm diameters and 100 µm away from each other were used to culture stem cells into spheroids. They created a micropatterned culture system to obtain and differentiate stem cell spheroids into osteogenic and adipogenic routes [44]. Micropatterned substrates are found to be particularly useful in neurogenic differentiation. Recknor et al. showed an alignment of rat hippocampal neural progenitor cells which in cocultures acquired neuronal morphology on polystyrene micropatterns with 16 μm groove width, 13 μm spacing, and 4 μm depth [45]. Garcia-Parra et al. demonstrated that micropatterned substrates (with repeating squares and rectangles with 300 × 300 μm^2^ and 300 × 400 μm^2^ areas) with neuronal ECM modifications induced neuronal differentiation [46]. In one study on myogenic differentiation, cells cultured and aligned on microchannels with 20 μm widths inhibited FAK-ERK signaling and differentiated the stem cells toward a myogenic phenotype [7]. In another study, microchannels with 20 μm widths differentiated human mesenchymal stem cells into myocardial lineage [47]. Induced pluripotent cells (h-IPSC) were shown to be differentiated on micropatterned substrates just like mesenchymal stem cells. Kusuma et al. showed endothelial differentiation of h-IPSCs on micropattern islands with 80–400 µm diameters and functionalized with several cell adhesion molecules [48]. Zhao et al. used micropatterned hydroxyapatite scaffolds to induce osteogenic differentiation of stem cells [49]. Overall, the products of photolithography and microfabrication technologies were shown to be able to direct cell fate. These prove the multifaceted nature of the technology and diverse applications of the products.

Previously, square pillar patterned substrates similar to those used in this study were used to characterize and measure cancer cell responses by our group and others [37,50,51,52,53,54]. This type of square pillars were shown to induce the deformation of nuclei, especially in cancer cells. It was deduced that quantification of the extent of deformations may help the detection of some phenotypical differences that exist between cancer cells. Low micron dimensions (≤10 µm) were shown to be more useful in this than the larger pattern features. Therefore, it was considered to be a good strategy to characterize cancer cell nuclear deformability using micro-nanofabrication methods and to study the phenotypical or molecular differences between different cancer cell types and between healthy and cancer cells.

Nuclear deformation of osteosarcoma cells was first observed by our group when micropatterned substrates were employed for tissue engineering applications [51]. This property of the patterned substrates was further investigated to show the effect of pillar size on the nuclear deformation of cancer cells [50]. It was shown that smaller micropatterns (≤8 µm) induced more prominent changes in nuclear shape. Later, the impact of the biomaterial [50] and the effect of surface hydrophobicity [55] on the nuclear deformation were also investigated. Hydrophobic surfaces and synthetic polymers with fewer functional groups that elicit fewer cell–material interactions evoked more pronounced nuclear deformations.

In conventional biological studies and cell culture, tissue engineering scaffolds and microfabricated substrates (in this case) are cultured in well plates. The samples are first placed in the well, which is followed by cell seeding and culture in an incubator. When analysis is required, samples are removed from the plate with help of a tool (e.g., tweezers) and transferred to the desired examination site. During this process, temperature and moisture cannot be controlled and immersing and removing samples result in shear forces on cells. There are several disadvantages of these methods: (i) low cell seeding accuracy and reproducibility, (ii) damaging cells during transfer, (iii) unsuitability for high-throughput imaging, (iv) no or little control over environmental parameters (temperature, moisture, etc.), (v) technical difficulties with live cell imaging, and (vi) high volumes of liquid required to process small samples due to large culture well volumes (treatment, growth media, dyes, etc.). The Ch-Pattern developed in this study aims to solve these problems by providing a small volume culture chamber, automation, suitability for high-throughput analysis, simplicity, faster analysis, and portability.

Here, we introduce a top load micropatterned chip that allows high-throughput microscopic and functional analysis of cancer cells that can be integrated to a microscope and molecular analysis workflow. This chip of poly(methyl methacrylate) (PMMA) was produced by microfabrication methods to be used with both upright and inverted microscopes. In this study, breast cancer cell lines (MCF-7 and MDA-MB-231) were chosen due to their distinct epithelial and mesenchymal invasiveness and metastatic properties as well as their biophysical properties [56]. MCF-7 cells show a more epithelial phenotype, stronger cell–cell contacts, and are less invasive and metastatic [57]. On the other hand, MDA-MB-231 is aggressive and metastatic with a spindle-like mesenchymal phenotype [58]. These two cell types, although originating from the same tissue and displaying a similar pathology, show different responses to microenvironmental cues [52,59].

The transparent micropatterned PMMA was the device in which MCF-7 and MDA-MB-231 were cultured. After seeding the cells onto the substrates, several live and fixed cell analyses (nuclear shape analysis, apoptosis, proliferation index, live dead, metabolic activity, etc.) were conducted. The chip allowed all the analyses, which included imaging, colorimetric, and molecular biological assays to be performed. By using these chip interfaces, we were able to study the differences between two cancer cell lines using imaging (deformation of cell nuclei) as well as cellular analyses (apoptosis, viability, proliferation). The microfabrication technology allowed us to fabricate the interface, and to study of cancer cells in vitro.

## 2. Materials and Methods

### 2.1. Preparation of Micropattern Bottom Chips

#### 2.1.1. Wafer Production

The micropillars were fabricated using a standard photolithography procedure. A silicon wafer (4-inch, University Wafer, South Boston, MA, USA) was used as the substrate and was spin-coated with a 10 μm thick layer of SU- 2100 (MicroChem) (1900 rpm, 45 s with a ramp rate of 500 rpm/s), baked (65 °C for 1 min, and 95 °C for 2 min) [50]. A custom photomask (Fineline Imaging, Colorado Springs, CO, USA) was used for exposing SU-8 to UV (I-line, 140 mJ/cm^2^ using a SUSS MA6 Mask Aligner). The wafer was baked at 65 °C for 1 min and 95 °C for 3 min and developed. Finally, the wafer was baked at 175 °C for 5 min [37,52,55].

#### 2.1.2. Production of PMMA Micropatterns

The wafer was copied to polydimethylsiloxane PDMS. PDMS was prepared by mixing Sylgard 184 and curing agent at a 1:10 ratio (*v*/*v*) (Dow Corning Company, Penarth, UK). PDMS was poured onto the micropillar wafers and cured under vacuum at 70 °C for 4 h. Cured PDMS was peeled from the wafer and used as the negative mold. Poly(methyl methacrylate) (PMMA) (Mw ≈120 kDa, Sigma, Neustadt, Germany) was prepared as a solution in chloroform (10% *w*/*v*) and poured into the PDMS molds, and the solvent was evaporated under the fume hood. These micropatterned PMMA films served as bottoms of the PMMA chips.

#### 2.1.3. PMMA Chip Production

The micropattern chips were fabricated using 4 mm thick PMMA sheets (BAS, Istanbul, Turkey) and 100 μm thick double-sided adhesive (DSA) film (ATM, Istanbul, Turkey) with a laser cutter (VersaLASER; Universal Laser Systems Inc., Scottsdale, AZ, USA). The dimension of each chip was 75 × 25 mm^2^, and each contained one to four wells with an 8 × 8 mm^2^ area for micropatterns (single-well, double-well, and multiwell chips). The size of the DSAs was 12 × 12 mm^2^ squares with 8 × 8 mm^2^ square holes.

#### 2.1.4. Chip Assembly

Micropatterned PMMA films were adhered to the bottom side of the PMMA chip using DSA (Supplementary Figure S1). Then, the chips were checked for liquid leakage and sterilized with UV (265 nm) for 15 min each side.

### 2.2. Characterization of PMMA Films

#### 2.2.1. Transparency of the Micropatterned PMMA

PMMA films were scanned in the 250–700 nm range by using a Multiscan UV Visible spectrophotometer (Thermo Scientific, Waltham, MA, USA). Films were examined with colorless growth media (DMEM High without phenol red). Wells containing only growth medium and no film served as the blank. Transmittance values were calculated.

#### 2.2.2. Scanning Electron Microscopy (SEM)

Samples were coated with Au under vacuum and examined by SEM (FEI Quanta 650 FEG) under high vacuum. Cell-seeded samples were air-dried after fixation with 4% paraformaldehyde (4% PFA), coated with Au using a sputter coater, and examined [37].

### 2.3. In Vitro Studies

Cell lines were obtained from commercial suppliers (MCF7, ATCC^®^ HTB-22^™^ and MDA MD 231, ATCC^®^ CRM-HTB-26^™^). MCF7 cells were cultured in DMEM low glucose (Biological Industries, Beit-Haemek, Israel) supplemented with 10% FBS, 100 U/mL^1^ penicillin, and 100 μg/mL^1^ streptomycin. MDAMB231 cells were cultured in DMEM (Biological Industries, Beit-Haemek, Israel) supplemented with 10% FBS, 100 U/mL^1^ penicillin, and 100 μg/mL^1^ streptomycin. Cells were grown in cell culture flasks until 90% confluency and detached using 0.05% Trypsin EDTA (Biological Industries, Israel). The cell suspension is counted and seeded on the micropatterns with a density of 5 × 10^4^ cells/mL^1^ (5 × 10^4^ cells/well), and the chips were cultured in a Petri dish at 37 °C, 5% CO_2_. For controls, the same number of cells was seeded into 24-well plates.

### 2.4. Deformation Analysis

#### 2.4.1. Fluorescence Microscopy

Cell-seeded samples were fixed in 4% paraformaldehyde, permeabilized with 1% Triton-X 100 solution (Applichem, Darmstadt, Germany), and incubated in bovine serum albumin (BSA) blocking solution (1%, *w*/*v*, in PBS) at 37 °C for 30 min. Then, films were incubated in Alexa Fluor 488^®^ labeled Phalloidin solution (1:50, in 0.1% BSA in PBS) for 1 h, 37 °C, and then DAPI (4′,6-diamidino-2-phenylindole) (0.1 µg/mL, in 0.1% BSA in PBS) (Invitrogen, Waltham, MA, USA) was used for 5 min at room temperature to stain the nuclei. Fluorescence micrographs were obtained with an upright fluorescence microscope (Zeiss Axio Imager M2, Jena, Germany). The micrographs were obtained by using the black and white filter of the microscope and pseudo-colored with magenta (actin) and cyan (nucleus) by the software system of the microscope.

#### 2.4.2. Digital Analysis of Nucleus Deformation

Fluorescence micrographs were analyzed by using the image analysis software ImageJ (NIH) to determine the deformations of the nuclei of cells by using five different shape descriptors: “Circularity”, “Feret”, “Roundness”, “Aspect Ratio”, and “Solidity” [60]. The equations of shape descriptors were presented in Supplementary Table S1. Shape analyses were performed using 100 cell nuclei from 5 images of each surface. Thresholding of images was done by auto local threshold analysis and the Phansalkar method [60].

### 2.5. Cell Viability and Apoptosis and Proliferation

#### 2.5.1. Live–Dead Analysis

The viability of cells on seeded films and tissue culture plates was determined with Live–Dead cell viability assay (Invitrogen, Waltham, MA, USA). After 24, 48, and 72 h culture, the medium was discarded, samples were washed three times with PBS, and they were double-stained with calcein (2 μM in PBS) and ethidium homodimer-1 (4 μM in PBS) (Molecular Probes, Eugene, Oregon, USA) for 5 min. After washing with PBS, samples were examined by fluorescence microscopy.

#### 2.5.2. DNA Quantification

PicoGreen Assay (Quant-IT PicoGreen dsDNA assay kit, Invitrogen, Waltham, MA, USA) is used for the quantification of DNA content. This assay determines the amount of DNA in the sample, from which cell number in the sample can be estimated. After culturing the cells on smooth and micropatterned PMMA for 24, 48, and 72 h, the cells were resuspended in 350 μL of RLT lysis buffer (RNeasy mini kit, Qiagen, Hilden, Germany). All samples were vortexed for 15 s and centrifuged for 1 min at 13,000 rpm. Then, 10 μL of the sample was diluted 10X in DNase-free water. Finally, 5 μL of this diluted sample was diluted in 195 μL of working buffer (199 μL of the Quant-IT dsDNA buffer and 1 μL Quant-IT dsDNA reagent prepared for each sample), after which the sample was shortly vortexed and incubated at room temperature for 5 min. Then, the DNA concentration was measured with the fluorometer by exciting at 485 nm and measuring the fluorescence intensity at 520 nm. All samples were prepared in triplicate. The linearity of the DNA measurements was evaluated for the RLT lysis buffer with a final buffer dilution of 400X·by preparing a calibration curve (Supplementary Figure S2). DNA standard concentrations in the measurement solution were 10, 25, 50, 100, 250, and 500 ng/mL.

#### 2.5.3. Caspase Assay

Caspase-3/7 assay was performed to determine apoptotic cell death with CaspaTag™ Caspase-3/7 In Situ Assay Kit, Fluorescein (Chemicon International, Temecula, CA, USA). Cell-seeded patterned and smooth films as well as Doxorubicin (0.2 µg/mL, 24 h) (Sigma, Burlington, MA, USA)-treated and untreated cell seeded tissue culture polystyrene (TCPS) were incubated with freshly prepared carboxyfluorescein-labeled fluoromethyl ketone peptide inhibitor of caspase-3 (FAM-DEVD-FMK) in culture medium at 37 °C under 5% CO_2_ for 1 h. Hoechst stain was used to stain the nuclei of the cells and incubated for 5 min at 37 °C under 5% CO_2_. Cells were rinsed with PBS and fixed with the fixative solution of the kit. Samples were observed under a confocal microscope (Zeiss, LSM800, Jena, Germany). The caspase-3/7 positive cell percentage was calculated using the Fiji program by using three micrographs for each sample.

#### 2.5.4. Annexin V Staining

Cells for apoptosis analysis were stained with fluorescein isothiocyanate (FITC)-Annexin V and PI (BD Biosciences, San Jose, CA, USA), using the FITC-Annexin V Apoptosis Detection Kit (BD Biosciences, San Jose, CA, USA) according to the manufacturer’s manual. Briefly, cell-seeded samples were washed twice with cold BioLegend cell staining buffer and then resuspended cells in Annexin V Binding Buffer at a concentration of 1 × 10^6^ cells/mL. Five μL of FITC Annexin V and 10 μL of PI solution were used, and the samples were incubated for 15 min at RT in the dark. Then, 100 μL of Annexin V Binding Buffer was added to each sample. Cell-seeded patterned and smooth films as well as doxorubicin (0.2 µg/mL, 24 h) (Sigma, Burlington, MA, USA) treated and untreated cell seeded TCPS were used for this analysis.

#### 2.5.5. Immunocytochemical Staining of Ki-67 Protein

Samples were prepared and stained for confocal microscopy. For Ki-67 and alpha-tubulin imaging, antibodies specific to these proteins (anti-Ki67 ab215226 antibodies and anti-alpha-tubulin ab28439, Abcam, Cambridge, UK) were used with 1:500 and 1:100 dilutions, respectively, at 4 °C overnight. These primary antibodies were tagged with Alexa Fluor 555 goat anti-rabbit and Alexa Fluor 488 goat anti-mouse at 1:100 dilutions, 37 °C, respectively. Finally, samples were stained with DAPI (0.1 µg/mL, 15 min, RT). Samples were imaged with Zeiss LSM 800 Confocal microscope with 405 nm, 488 nm, and 555 nm lasers. Afterward, images were analyzed using analysis software Fiji. For each analysis, three random regions were imaged for each sample, and three biological replicates were used for each group (total number of images analyzed per group = 9).

### 2.6. Real-Time Polymerase Chain Reaction (qRT-PCR)

MCF7 and MDAMB231 RNAs were isolated from cells using a Masterpure RNA Purification Kit (Epicenter, San Antonio, TX, USA), and RNA samples were DNAse-treated using a DNA-free™ DNA Removal Kit (Thermo Fisher Scientific, Waltham, MA, USA) according to the manufacturer’s instructions. For each group, 10 biological replicas (10 films seeded with MCF7 or MDAMB231 cells) were used. Each RNA sample was run three times (technical replica). Then, 1 μg RNA from each sample was converted to cDNA using a RevertAid First Strand cDNA Synthesis kit (Thermo Fisher Scientific, Waltham, MA, USA). RNA concentrations were measured by a Nanodrop 2000 C (Thermo Scientific, Waltham, MA, USA). Primers for *GAPDH* (glyceraldehyde-6-phosphate dehydrogenase) were selected using NCBI Primer-BLAST using accession number NM_001289746.1 (Supplementary Table S2) [61]. Primers for *CDKN1A* (Cyclin-Dependent Kinase Inhibitor 1A, p21), *CCNA2* (Cyclin A2), and *CCNB* (Cyclin B) were selected using NCBI Primer-BLAST using accession numbers NM_078467.2, NM_001237.3, and NM_031966.3, respectively (Supplementary Table S2) [62,63,64]. Primers for *Ki-67* (Marker of Proliferation Ki-67) were selected using NCBI Primer-BLAST using accession number NM_002417.4 (Supplementary Table S2) [65]. First-strand cDNA synthesis via RT-PCR was performed with 1 μg RNA from each sample with a RevertAid First Strand cDNA Synthesis Kit (Thermo Fisher Scientific, USA) and a thermal cycler (iCycler, BIO-RAD, Hercules, CA, USA) with the oligo (dT)_18_ primers supplied with the kit. The reverse transcription step ran for 60 min at 42 °C, which was followed by reaction termination for 5 min at 70 °C. Real-time quantitative PCR was performed using qPCR Master Mix (Promega, Madison, WI, USA) under the conditions 2 min 95 °C (HotStarTaq activation), 30–40 cycles of 15 s denaturation at 95 °C, and 60 s annealing/extension at 60 °C followed by melting. For each reaction, 50−0.005 ng cDNA sample was run according to the manufacturer’s directions. For the evaluation of isolation quality of RNA samples, RNA, and for primer quality, PCR products of the target gene were run on 1% and 2% agarose gel, respectively. For negative controls, non-template and no reverse transcription reactions were performed. All qRT-PCR results were normalized to the unpatterned PMMA values using fold changes (Supplementary Figure S3). The ∆∆CT method was used for normalization. *GAPDH* was selected as the housekeeping genes (HKG). All PCR data from micropatterns and unpatterned controls were normalized to the *GAPDH* using the following formula:

(1) $$\Delta CT=CT\left(target\right)-CT\;\left(GAPDH\right).$$

Afterward, micropatterned samples were normalized to *unpatterned* samples:

(2) $$\Delta \Delta CT=\Delta CT\left(micropatterned\right)-\Delta CT\left(unpatterned\right).$$

The results were presented in the form of fold change (2^−∆∆*CT*^) in relation to the *unpatterned* control group, where the value of the control group is always 1, values >1 upregulated genes, and values <1 downregulated genes.

### 2.7. Statistical Analysis

All quantitative data in this study were expressed as mean ± standard deviations with *n* ≥ 2 unless otherwise stated. Shape analyses were performed using 100 cell nuclei from 5 images of each surface. A normality test on all collected data was performed by the Shapiro–Wilk test. Statistical analysis was performed by one-way or two-way ANOVA (analysis of variance) test followed by Tukey’s test for normally distributed data and a Kruskal–Wallis test for non-normally distributed data. Normally distributed data were presented as symbol plots, where symbols and error bars represent the mean and standard deviation, respectively. Non-normal data are presented as box–whisker plots, where boxes represent 25th and 75th percentiles and whiskers represent the values from min to max. The line in the middle of the box is plotted at the median and “+” is plotted at the mean. Statistical significance is set at a 95% confidence level for all tests (*p* < 0.05).

## 3. Results and Discussion

### 3.1. Fabrication and Characterization of Transparent, Micropillar-Bottomed Culture Chip (Ch-Pattern)

This study aimed to produce a transparent bottomed chip decorated with micropatterns to study cell activity which otherwise is hard to follow and analyze because they require direct imaging or analysis. In previous studies on micropatterned substrates, films or scaffolds were used in well plates in conventional tissue culture setups [66,67,68,69]. We designed a PMMA chip with a micropatterned bottom to avoid all these and performed analyses with live and fixed cells involving imaging and lysis for DNA or RNA downstream investigations. With this design, cell–substrate interactions could be traced without interference of human manipulation of the substrate. The water contact angle (WCA) of the smooth PMMA was around 89°, while that of the patterned film was 132°. These results were previously obtained and reported by our group. The increased hydrophobicity made it hard for cells to adhere and amplified the deformability of the cells [52].

**Figure 1:**
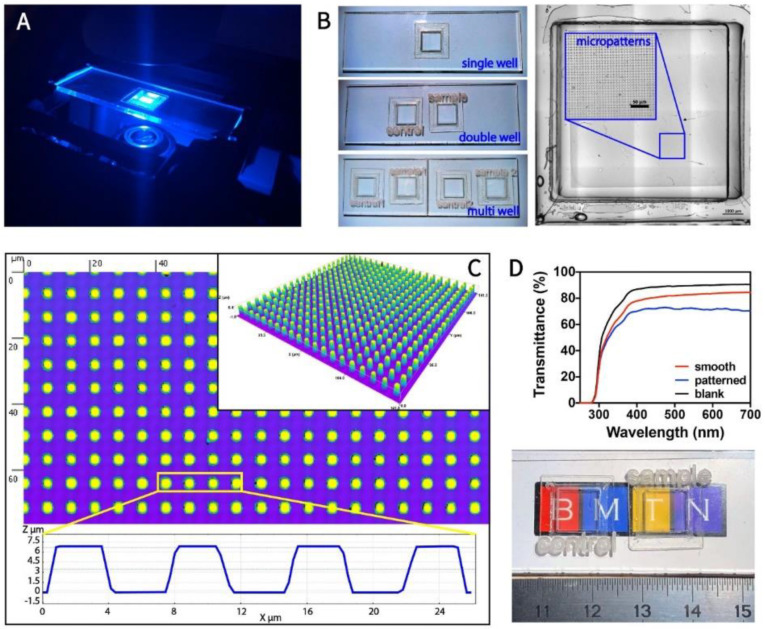
Images and characterization of transparent, micropillar-bottom culture chip (Ch-Pattern)**.** (**A**) CLSM image of the Ch-Pattern. (**B**) Organization of the single-, double-, and multi-well chips for the single and simultaneous imaging for selected applications (**left panel**); stitched phase-contrast image of the chip (**right panel**). (**C**) Surface profile of the micropatterns obtained using a profilometer. (**D**) Transparency of the PMMA micropatterned substrate. The transmittance of the smooth and patterned substrates was measured against blank.

Supplementary Figure S4 shows the fabrication of a PMMA chip that has micropatterned (Ch-Patterned) or smooth (Ch-Smooth, control) PMMA bottoms. Figure 1 shows the design, topography, and transparency of the chip used in this study. Imaging of the live and fixed cells was performed using an inverted confocal microscope (Figure 1A). The chips used consisted of single, double, and multiwells depending on the purpose; double and multiwell were used to compare the cell behavior on smooth and patterned bottoms and in the study of two cell lines simultaneously (Figure 1B). Ch-Patterned chips were characterized with transmitted light detector (ESID) of the confocal microscope using an automated stage and by stitching multiple images together (Figure 1B). Since the wells on the chips were at defined locations on the stage, automatization by Zen Blue software of the microscope was possible. The whole chip bottom could be scanned, and selected regions could further be imaged at a higher magnification according to the coordinates obtained from the complete scan. In this study, the pillar dimensions were: 4 × 4 µm^2^ area, 4 µm gap, and 7 µm height. PMMA surfaces were produced on the PDMS copies of the original silicon wafers using solvent casting. PMMA surfaces were characterized for replication accuracy using a profilometer (Figure 1C). The pillar area, height, and gaps were measured as 4 × 4 µm^2^, 4 µm, and 6.75 µm, respectively. The replication fidelity was found to be similar to that reported in our previous study [70]. The transparency of the micropatterned PMMA at 250–700 nm was measured (Figure 1D). Both smooth and micropatterned PMMA were found to be highly transparent in the whole range (transmittance > 70%). These make the design and the material choice highly suitable for live cell imaging and cell–material interaction studies using an inverted microscope.

### 3.2. Imaging and Nuclear Shape Analysis of Breast Cancer Cells on Micropillar-Bottomed Culture Chip (Ch-Pattern)

**Figure 2:**
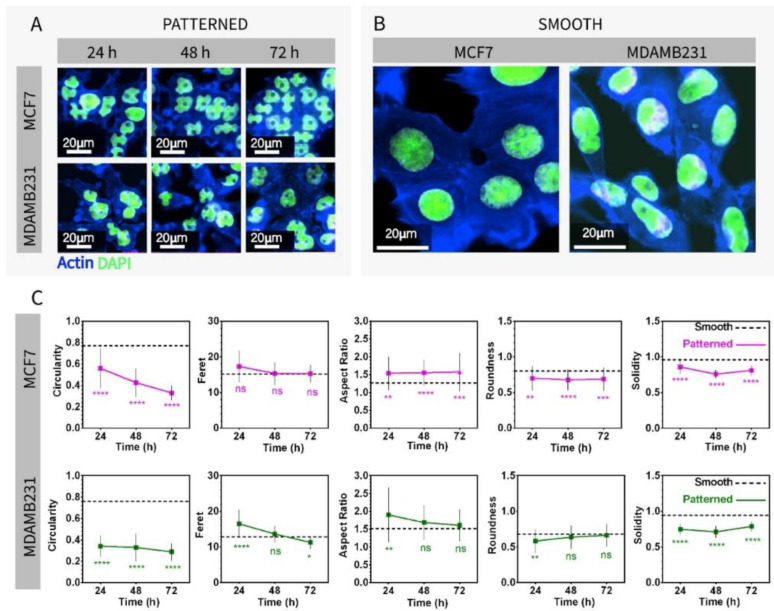
Micrographs of the MDAMB231 and MCF7 cell cytoskeleton and nuclei, and the nuclear deformations measured from these images. (**A**) Micrographs of the breast carcinoma cells on micropatterned chips imaged at 24, 48, and 72 h of cell culture (scale bars: 20 µm, blue: Alexa Fluor Phalloidin for F-actin, green: DAPI for DNA). (**B**) Micrographs of the breast carcinoma cells on smooth chip imaged at 24 h of cell culture (scale bars: 20 µm, blue: Alexa Fluor Phalloidin for F-actin, green: DAPI for DNA). (**C**) Nuclear shape analysis of MDAMB231 and MCF7 cells from the micrographs of micropatterned and smooth chips. Circularity, Feret, Aspect Ratio, Roundness, and Solidity parameters were used for the analysis (one-way ANOVA. MCF7: *p*_circularity_ < 0.0001, *p*_feret_ = 0.0252, *p*_aspect ratio_ < 0.0001, *p*_roundness_ < 0.0001, *p*_solidity_ < 0.0001. MDAMB231: *p*_circularity_ < 0.0001, *p*_feret_ < 0.0001, *p*_aspect ratio_ = 0.0155, *p*_roundness_ = 0.0152, *p*_solidity_ < 0.0001. * *p* < 0.005, ** *p* < 0.001, *** *p* < 0.0005, **** *p* < 0.0001).

PMMA chips create a controlled environment for imaging both live and fixed cells and for staining. It requires a minimal amount of antibody solutions (around 50 µL) due to its design and small chambers, which is an advantage for minimizing the use of costly antibodies and media. In addition, it allows automatized imaging of the surfaces and enables obtaining multiple images from the same location of different surfaces with fixed dimensions (five separate images were obtained for each surface). On Ch-Pattern and Ch-Smooth, two different breast cancer cells, MCF7 (noninvasive, malignant) and MDAMB231 (invasive, malignant), were cultured. Cells were stained while in the chambers (Figure 2). The cells were imaged with an inverted confocal microscope equipped with an environmental chamber on patterned (Figure 2A) and smooth (Figure 2B) chips. Additionally, chips were imaged with scanning electron microscope (SEM) (Supplementary Figure S5). In both confocal images and the SEMs, cells on Ch-Smooth showed larger spread than on Ch-Pattern. Shape analyses of nuclei of the cells were performed with image analysis software ImageJ (NIH) using confocal micrographs. Micropatterned surfaces morphologically distorted cells, which was reported in several studies in the last decade [5,52,55,59,71,72,73]. Both malignant breast cancer cells were highly deformed on the Ch-Pattern (Figure 2A) but not deformed (remained elliptical) on Ch-Smooth (Figure 2B). Five different shape descriptors were used to analyze the shape distortions quantitatively: Circularity, Feret, Aspect Ratio, Roundness, and Solidity (equations are provided in Supplementary Table S1). Statistical significance of the results was determined by comparing the shape distortions on the patterned surface with those on the smooth. Culture duration had different effects on the two cell lines: In MCF7 cells, Feret on patterned surfaces was not significantly different than the smooth surfaces through 72 h of incubation. Circularity decreased significantly in time, which indicates increasing deformation of the cell nuclei with time. The Aspect Ratio was significantly higher, whereas Roundness and Solidity were significantly lower on patterned surfaces. However, these three descriptors did not change with time. On the other hand, with MDAMB231 cells, Feret, Aspect Ratio, and Roundness on Ch-Pattern showed significant differences with time while Circularity and Solidity on Ch-Pattern were not affected by time and were always much lower than the smooth. Changes in the shape descriptors with time except Circularity were similar in two different cell lines. When Circularity on Ch-Patterned was compared with on Ch-Smooth, MDAMB231 cells showed significantly lower Circularity at the very beginning of the culture time, whereas MCF7 cells had Circularity that decreased over time. Nuclear deformation has been reported as a continuous process (can be observed even after the cell division as long as the cells are on the same topography) [50,74] and increases over time [52,53].

### 3.3. Testing the Metabolic Activity and Proliferation of Cells on Micropillar Bottom Culture Chip (Ch-Pattern)

**Figure 3:**
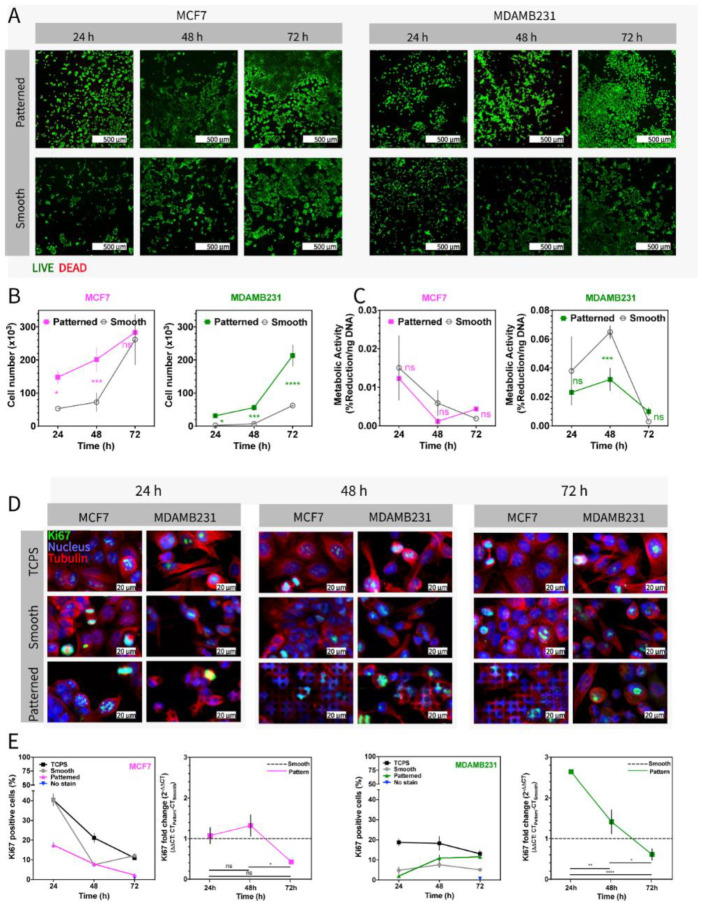
Live–Dead, cell number, metabolic activity, proliferation index (Ki-67 positive cell ratio) and Ki-67 gene expression of the MCF7 and MDAMB231 cells on Ch-Pattern and Ch-Smooth chips. (**A**) Live–Dead assay of MDAMB231 and MCF7 cells cultured on Ch-Pattern and Ch-Smooth for 72 h (Scale bar: 500 µm, green: Calcein—Live, red: Ethidium Bromide—Dead). (**B**) Proliferation of MDAMB231 and MCF7 cells on Ch-Pattern and Ch-Smooth at 72 h of culture. Concentration was measured using PicoGreen DNA quantification assay, and each result was converted to cell number by dividing total content by the average DNA content per cell. (Two-way ANOVA, MCF7: *p*_time_ < 0.0001, *p*_sample_ = 0.0015, MDAMB231: *p*_time_ < 0.0001, *p*_sample_ < 0.0015; * *p* < 0.005, ** *p* < 0.001, *** *p* < 0.0005, **** *p* < 0.0001). (**C**) Metabolic activity of MDAMB231 and MCF7 cells on Ch-Pattern and Ch-Smooth (72 h culture). Alamar blue reduction amount was normalized to DNA content. (Two-way ANOVA, MCF7: *p*_time_ < 0.0001, *p*_sample_ = 0.4236, MDAMB231: *p*_time_ < 0.0001, *p*_sample_ = 0.0181; * *p* < 0.005, ** *p* < 0.001, *** *p* < 0.0005, **** *p* < 0.0001). (**D**) CLSM micrographs of two cells on Ch-Pattern and Ch-Smooth showing Ki-67 and α-tubulin staining (Scale bar: 20 µm, green: Ki-67, red: α-tubulin, blue: DAPI). (**E**) Proliferation index (Ki-67 positive cell percentage) of cells on TCPS, Ch-Pattern, Ch-Smooth, and no stain control and Ki-67 gene expression of the cells on Ch-Pattern and Ch-Smooth (Proliferation index: Two-way ANOVA, MCF7: *p*_time_ < 0.0001, *p*_sample_ < 0.0001, MDAMB231: *p*_time_ = 0.0468, *p*_sample_ < 0.0001, see Supplementary Figure S6 for statistical analysis. Ki-67 PCR: One way ANOVA, MCF7: *p* = 0.0323, MDAMB231: *p* = 0.0001; * *p* < 0.005, ** *p* < 0.001, *** *p* < 0.0005, **** *p* < 0.0001).

The design of the chip makes volumes as small as 50 µL possible to cover the bottom, and multiwell chips allow analysis of multiple markers simultaneously. Moreover, cells could be cultured on the chip and directly moved to the microscope stage without moving the scaffolds. Cells are subjected to minimal change of temperature and pH during the transfer between the laboratories. In Figure 3, on Ch-Pattern and Ch-Smooth, the metabolic activity and proliferation of the two cell lines were evaluated with several different methods. Live–Dead assay of the cells shows that both cell lines had high viability after 72 h in culture on both patterned and smooth PMMA surfaces (Figure 3A). It can be deduced that there was no detrimental effect of the polymer or the micro features on cell behavior. The cells were less crowded on smooth surfaces than on patterned surfaces, possibly because the initial attachment on patterned surfaces was always higher probably due to the high surface/volume ratio on the microfeatures and the presence of edges for cells to adhere. DNA amounts of the cells were calculated with PicoGreen DNA quantification assay (Figure 3B). Cells were lysed on chip, and the lysate was used for DNA quantification. On-chip lysis allowed control over the procedure as well as decreased the reagent quantities without decreasing the concentrated collection of DNA. Total DNA contents were divided to average the DNA content per cell to determine the cell number. The MCF7 cell number increased linearly in time on the patterned surfaces, whereas MDAMB231 cells showed a delayed increase in cell number after 48 h. This difference between the two cell lines may arise from the doubling times, which are reported by ATCC as 29 h and 38 h for MCF7 and MDAMB231, respectively. While the numbers of the two cell lines increased in time, they did not show the same rate of increase in their metabolic activities (Figure 3C). MCF7 showed a slow rate of decrease in metabolic activity through 72 h on both Patterned and Smooth. There was no significant different between the metabolic activities of cells on Patterned or Smooth. MDAMB231 cells showed a slight increase but eventually showed a decrease in metabolic activity. At 48 h, cells on Smooth had significantly higher metabolic activity than on Patterned. Similarly, the Ki-67 fold change was decreased for MDAMB231 cells on Patterned, even though this change was not reflected in the Ki-67 positive cells yet (Figure 3E). This might be due to the time required for protein synthesis and degradation needed for the reflection of mRNA changes. Although the duration of observation was 72 h in this study, this change may be reflected later. The metabolic activity of the cells was measured with Alamar Blue assay on chip, and the percent reduction values of the dye were divided by DNA content (ng) to normalize the results with the amount of DNA of each cell. Graphs show that metabolic activities decreased when the proliferation rate increased. Proliferation marker Ki-67 was determined using immunocytochemistry and RT-qPCR simultaneously. Cells were either fixed and stained on chip or lysed, and RNA was isolated. Figure 3D shows the staining of cells with a Ki-67-specific protein marker (green) on Ch-Pattern, Ch-Smooth, and on TCPS as controls. Cells were also stained with DAPI (blue) and α-tubulin (red) to visualize their nuclei and microtubules, respectively. Micrographs show the Ki-67 positive cells in green, and the number of these Ki-67 positive cells were counted from the micrographs of the chips using automatized stage (Figure 3B). The results show that in MCF7, Ki-67-positive cells decreased over time, whereas in MDAMB231, the number stayed almost the same on smooth and TCPS surfaces and increased on the patterned surface through the culture duration. On the other hand, RT-qPCR results show that Ki-67 gene expression decreased in both cell lines (Figure 3E). In summary, all tests show that micropatterned surfaces led to decreased metabolic activity of the cells in contrast to increasing cell number. It has been reported that nano and micro-scale surface topographies can affect cell proliferation, and it was shown that the proliferation rate increased on microstructures possibly due to the increasing number of attachment sites on micropatterned surfaces [75,76,77]. However, decreased metabolic activity could result from the nature of PMMA itself, which is known to arrest cells at the stationary (G0/G1) phase of the cell cycle [78,79]. Moreover, it was also reported that deformations in the nuclei of the cells led to the senescence of the cells especially when the nuclear abnormalities were originated from Lamin A/C-related genes and proteins [80].

**Figure 4:**
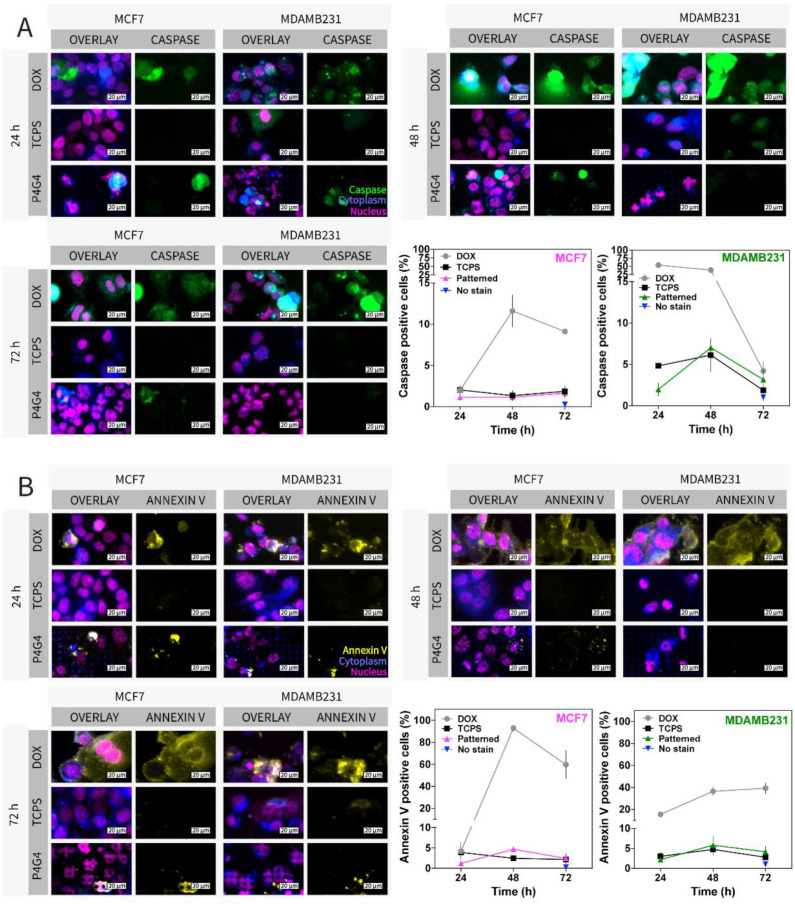
Apoptosis assay of MCF7 and MDAMB231 cells on Ch-Pattern and TCPS. (**A**) Caspase 3/7 activity of the cells was detected using CaspaTag (FAM-DEVD-FMK) on Ch-Pattern, and doxorubicin was used as the inducer of apoptosis (positive control) and TCPS as negative control (Scale bar: 20 µm, green: Caspa-Tag-Caspase 3/7, blue: CellTracker-Cytoplasm, and pink: Hoechst). Caspase-positive cell percentage was calculated from caspase-positive cells and the total number of cell nuclei (two-way ANOVA, MCF7: *p*_time_ = 0.0013, *p*_sample_ < 0.0001, MDAMB231: *p*_time_ < 0.0001, *p*_sample_ < 0.0001, see Supplementary Figure S7). (**B**) Annexin V protein on the surface of the cells was detected using Annexin V antibody on Ch-Pattern, and doxorubicin was used as the inducer of apoptosis (positive control) and TCPS as negative control (scale bar: 20 µm, yellow: Annexin V, blue: CellTracker-Cytoplasm, and pink: DAPI). Annexin-positive cell percentage was calculated from Annexin V-positive cells and the total number of cell nuclei (two-way ANOVA, MCF7: *p*_time_ = 0.0014, *p*_sample_ < 0.0001, MDAMB231: *p*_time_ < 0.0001, *p*_sample_ < 0.0001, see Supplementary Figure S8).

We also investigated whether micropatterns affect apoptosis (Figure 4). Cells were seeded on TCPS and treated with doxorubicin (DOX) (positive control) and used without DOX treatment (negative control). Caspase-3/7 and Annexin V were used to determine apoptosis in live cells. Caspase-3/7 is a protease and plays a role in the cleavage of cysteine and aspartic acid amino acids during apoptosis in cells; thus, the presence of Caspase-3/7 in cells shows an apoptotic activity [81]. Annexin V, on the other hand, is specific to phosphatidylserine on the cell membrane, which is a phospholipid transported from the inner leaflet to the outer leaflet of the cell plasma membrane with pro-apoptotic signals at the early stage of apoptosis and helps phagocytes recognize apoptotic cells [81,82]. In the confocal micrographs (Figure 4A,B), only DOX-positive control groups showed caspase or Annexin V-positive cells at high numbers. DOX is a cytotoxic agent and was used as a positive control in this experiment. It induces cell death and apoptosis fast; hence, there is a steep increase in apoptotic cells in the first 48 h of the experiment. Positive cells in the micrographs were counted, and the proportion of Caspase and Annexin V-positive cells were calculated and normalized to the total number of cells. It was seen that numbers of apoptotic cells were very low on drug-free patterned and TCPS surfaces when compared with DOX-treated surfaces (Figure 4A,B). Thus, in both assays, micropatterned surfaces did not induce apoptosis in the cells. In the literature, however, it was shown that mechanotransduction can affect the apoptotic behavior of the cells [83,84]. For example, it was reported that fibroblasts subjected to mechanical stimuli showed increased apoptosis and mechanotransduction-related genes (lamin and emerin) [83]. In another study involving micropattern islands, cells showed higher apoptotic behaviour on smaller-sized islands compared to larger surface area islands possibly because cells had smaller spreading areas on small sized islands [84]. However, a direct effect of micropatterns on apoptosis has never been reported in the literature. Here, our results show that the deformation of the cells do not induce apoptosis in breast cancer cells.

**Figure 5:**
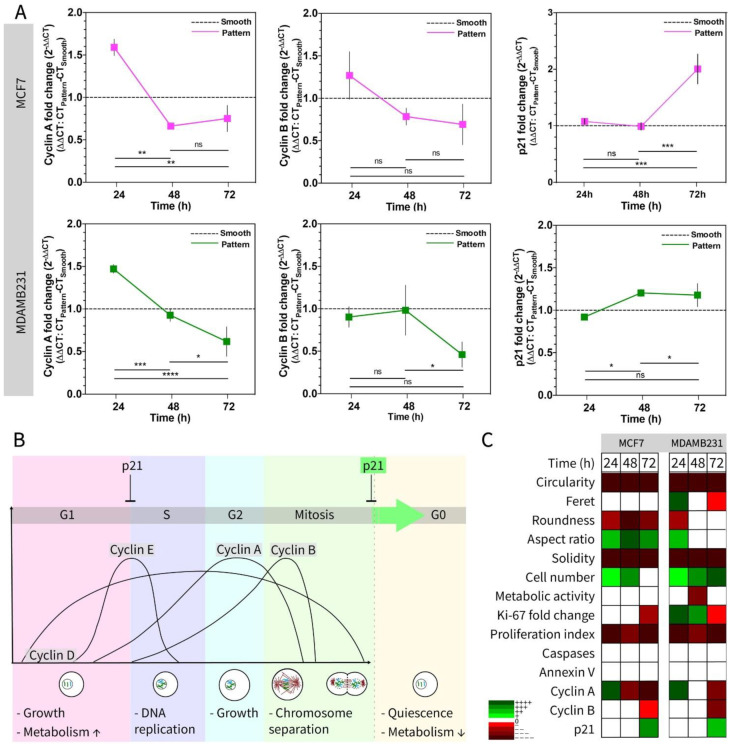
Cell cycle gene expression, scheme, and heatmap of the all metabolic and proliferative parameters of the MCF7 and MDAMB231 cells analyzed on Ch-Pattern at 24, 48, and 72 h of culture. (**A**) Cyclin A, B, and p21 expression of the MCF7 and MDAMB231 cells on Ch-Pattern. Results are normalized to Ch-Smooth and GAPDH expression (*∆∆CT*, see Materials and Methods (Heading 2)) (one-way ANOVA, MCF7: *p*_CyclinA_ < 0.0001, *p*_CyclinB_ = 0.0073, *p*_p21_ < 0.0001; MDAMB231: *p*_CyclinA_ < 0.0001, *p*_CyclinB_ = 0.0005, *p*_p21_ = 0.0027; * *p* < 0.005, ** *p*< 0.001, *** *p* < 0.0005, **** *p* < 0.0001). (**B**) Schematic of the cell cycle control genes. (**C**) Heatmap of the metabolic activity, image, and PCR analyses of the MCF7 and MDAMB231 cells cultured on the chips for 24, 48, and 72 h. The heatmap is constructed using statistical analysis data of the Ch-Pattern compared to Ch-Smooth. (* *p* < 0.005, ** *p* < 0.001, *** *p* < 0.0005, **** *p* < 0.0001; +/green: increase/upregulation, −/red: decrease/downregulation, white: no significant change).

Figure 5 shows the effect of micropatterned surfaces on the progression of the cell cycle in breast cancer cell lines through RT-qPCR analysis (Figure 5A), a scheme summarizing the cell cycle control by the cyclins (Cyclin A and B) and cyclin-dependent kinase inhibitor protein (p21) (Figure 5B), and a heat map of proliferative and metabolic activity-regulated molecules tested in this study (Figure 5C). Gene expression of cell cycle regulatory proteins Cyclin A, Cyclin B, and p21 were determined with RT-qPCR to study the effect of micropatterned surfaces on the cell cycle regulation of the two breast cancer cells (Figure 5A). It is known that p21 interacts with Cyclin A and Cyclin B at the later stages (G2/M) of the cell cycle, and the upregulation of p21 promotes arrest in the G2/M phase of the cell cycle [85]. In addition, the upregulation of cyclin A and B promotes entry into the M phase and regulates mitosis [86]. Our results show that cyclin A and B decrease, whereas p21 increased in time. This result is coherent with the proliferation and metabolism results (Figure 3) showing the decrease in metabolic activity of the cells. Two breast cancer cell lines (noninvasive malignant MCF7 and highly invasive malignant MDAMB231) were compared using viability, metabolism, proliferation, and apoptosis as well as shape changes, and a time-dependent heat map was constructed (Figure 5C). In the heat map, all the results were presented in comparison to smooth surfaces (green means increase or upregulation, red shows decrease or downregulation, and white boxes show no significant change). The map shows that among the five shape descriptors (Circularity, Feret, Roundness, Aspect Ratio, Solidity), Circularity and Solidity are the most distinctive descriptors to distinguish the nuclear deformation on Ch-Pattern from Ch-Smooth. When the proliferation, metabolic activity, and apoptosis of the cells on Ch-Pattern and Ch-Smooth were compared, it was observed that cell numbers were high and increased very significantly on patterned surfaces, and it was more distinct in MDAMB231 cells. Meanwhile the proliferation index was significantly lower on patterned surfaces, which needs further investigation of the cell cycle progression on these micropatterns. The apoptotic behavior of the cells was the same on both patterned and smooth surfaces, and cell cycle regulation did not show a consistent difference between patterned and smooth surfaces.

## 4. Conclusions

In conclusion, this study shows that the PMMA chip decorated with micropatterns at the bottom can provide a controlled 2.5D environment for cell culture and can be used to assess cancer cell behavior. The chip has the following advantages over the conventional cell culture methods and imaging systems: (i) precise and simultaneous control of microenvironment, (ii) easy to build, (iii) reproducible cell seeding, (iv) high-throughput imaging, (v) live cell imaging, and (vi) small volume of reagents used in analysis. All these properties contribute to the simplicity and versatility of the transparent, micropillar bottom chip. The control over the many parameters affecting the cell behavior such as temperature, oxygen, pH, and medium volume could be maintained in this chip in a more convenient manner. The microscale design of the chip has the advantage of controlling these parameters in a precise and more effective way compared to larger sizes. Even though it lacks the complexity of most of the sophisticated organ-on-a chip microfluidics devices, the simplicity allows easy production and use by multiple researchers with minimal training on microfabrication techniques. Here, we showed that this simple chip design allows multiple analyses (morphological, live–dead, Alamar blue, apoptosis, and PCR) of cancer cells from two different cell lines temporally with more than fifty samples in each group with at least five images of them. This adds up to hundreds of chips and millions of cells analyzed in total, showing the high-throughput capacity of the simple design.

In addition, the results show that micropatterned surfaces can amplify the deformability properties of cancer cells originate from the changes in mechanotransduction pathways. These surfaces designed with micropatterns do not result in an apoptotic fate for the cells and do not hinder the proliferation of cells but induce cell senescence. Thus, micropatterned surfaces can be optimal surfaces for cell cycle regulation studies in cancer cells, which are known to be regulated differently than the healthy cells. The effects of micropatterns on cell cycle need further investigation.

The device presented here allows discrimination of cancer cells using their morphology with micron-level precision and quantification of these properties that are otherwise not possible. It also allows good environmental control and protection for the samples proving itself an excellent tool to study cellular properties using their responses to micro cues as well to screen cellular differences live on chip.

## Supplementary Materials

The following supporting information can be downloaded at: https://www.mdpi.com/article/10.3390/mi13010093/s1, Figure S1: Chip and micropattern design; Figure S2: DNA quantification calibration curve; Figure S3: Calibration curves of the primers used for RT-qPCR; Figure S4: Micropatterned surface and chip preparation scheme; Figure S5: SEM images of MCF7 and MDAMB231 cells on Ch-Smooth and Ch-Pattern chips; Figure S6: Ki67 proliferation index of MCF7 and MDAMB231 cells. (Two way ANOVA, MCF7: *p*_time_ < 0.0001, *p*_sample_ < 0.0001, MDAMB231: *p*_time_ = 0.0468, *p*_sample_ < 0.0001); Figure S7: Caspase analysis of MCF7 and MDAMB231 cells (Two way ANOVA, MCF7: *p*_time_ = 0.0013, *p*_sample_ < 0.0001, MDAMB231: *p*_time_ < 0.0001, *p*_sample_ < 0.0001); Figure S8: Annexin V analysis of MCF7 and MDAMB231 cells (Two way ANOVA, MCF7: *p*_time_ = 0.0013, *p*_sample_ < 0.0001, MDAMB231: *p*_time_ < 0.0001, *p*_sample_ < 0.0001); Table S1: Shape descriptors and their equations; Table S2: Primer forward and reverse sequences used for qRT-PCR experiments, their NCBI accession numbers and their references.
