# Supplementary material for: A Cell Culture Chip with Transparent, Micropillar-Decorated Bottom for Live Cell Imaging and Screening of Breast Cancer Cells
Source: Micromachines (Basel). 2022 Jan 7;13(1):93. doi: 10.3390/mi13010093 (PMC8779566; doi:10.3390/mi13010093)
Supplement: Supplementary file 1 [file micromachines-13-00093-s001.zip › micromachines-1543615-supplementary.pdf]

A Cell Culture Chip with Transparent, Micropillar-Decorated Bottom for Live Cell Imaging and Screening of Breast Cancer Cells

Menekse Ermis [1,†], Ezgi Antmen [1,†], Ozgur Kuren [1], Utkan Demirci [2] and Vasif Hasirci [1,3,4,*]

[1] BIOMATEN, Center of Excellence in Biomaterials and Tissue Engineering, Middle East Technical University, Ankara 06800, Turkey; e.menek@gmail.com (M.E.); ezgiantmenn@gmail.com (E.A.); ozgur.kuren97@gmail.com (O.K.)
[2] Canary Center for Cancer Early Detection, Department of Radiology, Electrical Engineering Department, Stanford University, Palo Alto, CA 94305, USA; utkan@stanford.edu
[3] Department of Medical Engineering, Acibadem Mehmet Ali Aydinlar University, Istanbul 34684, Turkey
[4] ACU Biomaterials Center, Acibadem Mehmet Ali Aydinlar University, Istanbul 34684, Turkey
* Correspondence: vasif.hasirci@acibadem.edu.tr
† These authors contributed equally to this work.

Supplementary Information

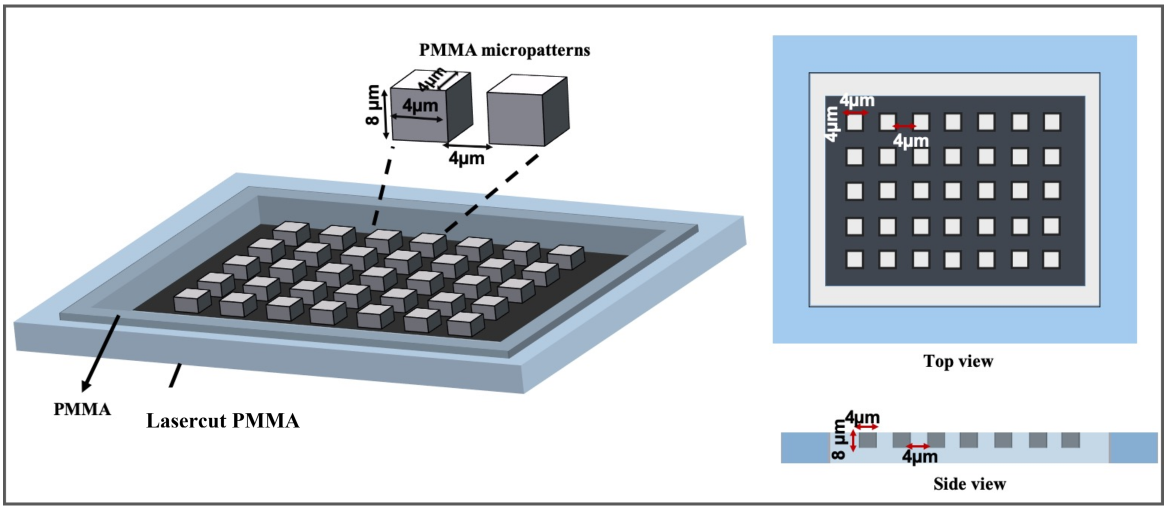

Supplementary Figure S1. Chip and micropattern design.

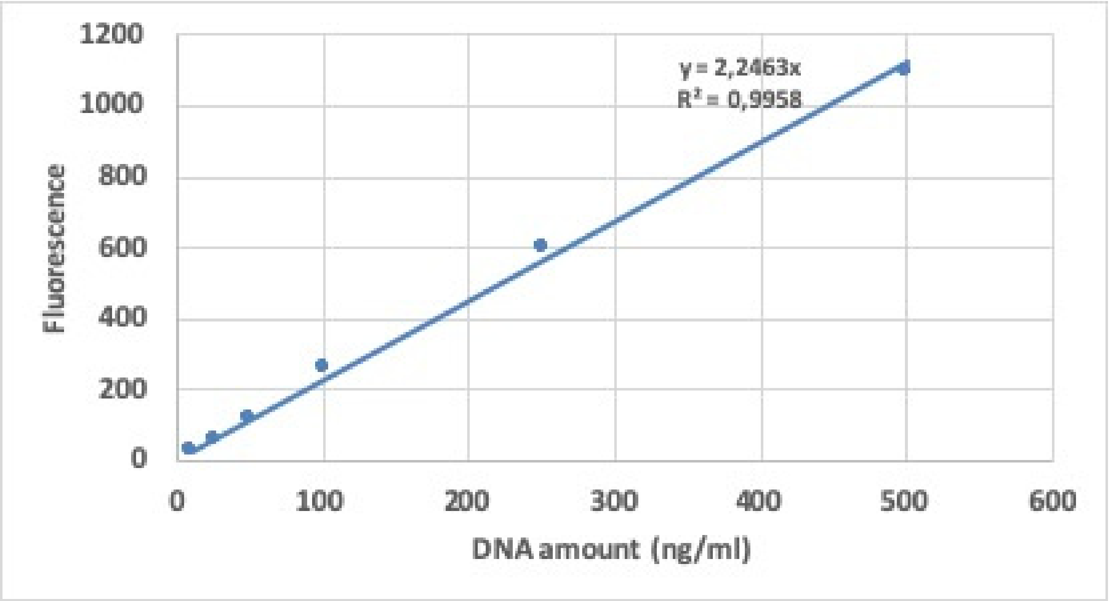

Supplementary Figure S2. DNA quantification calibration curve.

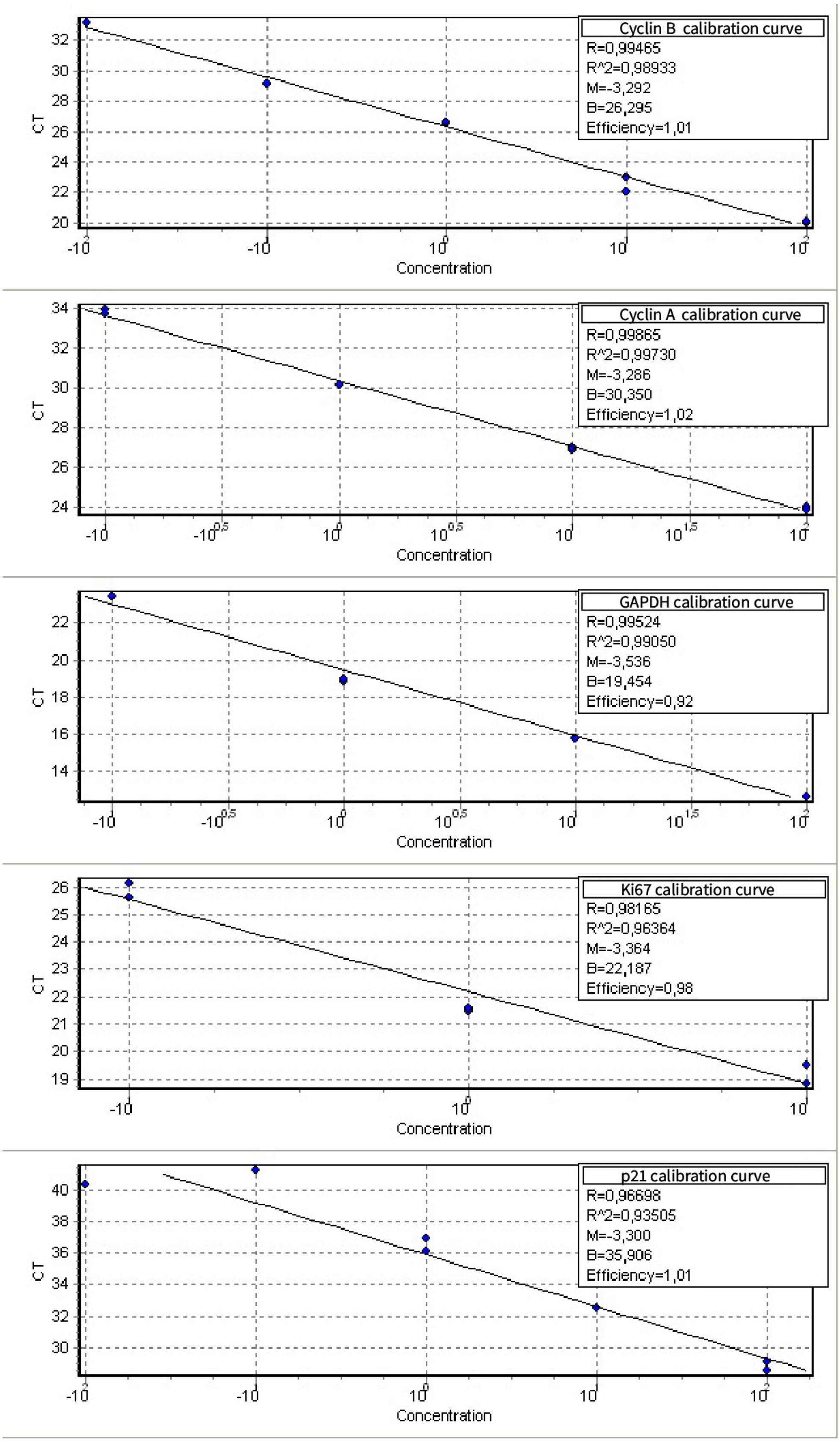

Supplementary Figure S3. Calibration curves of the primers used for RT-qPCR.

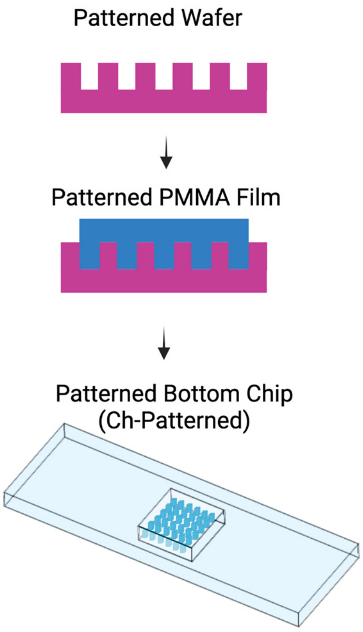

Supplementary Figure S4. Micropatterned surface and chip preparation scheme.

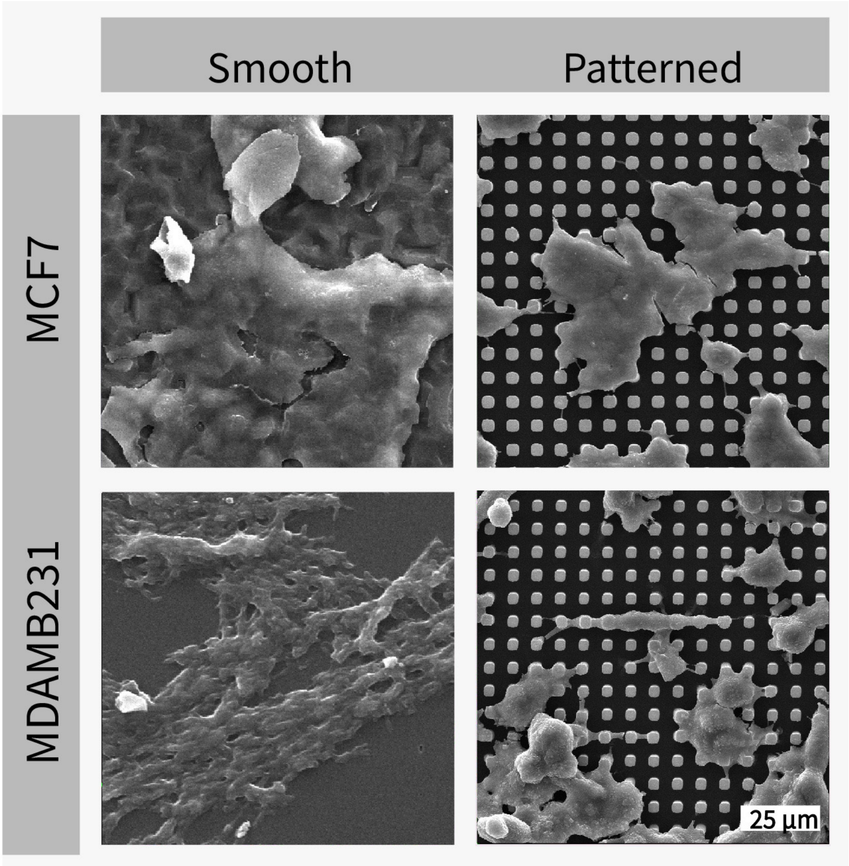

Supplementary Figure S5. SEM images of MCF7 and MDAMB231 cells on Ch-Smooth and Ch-Pattern chips.

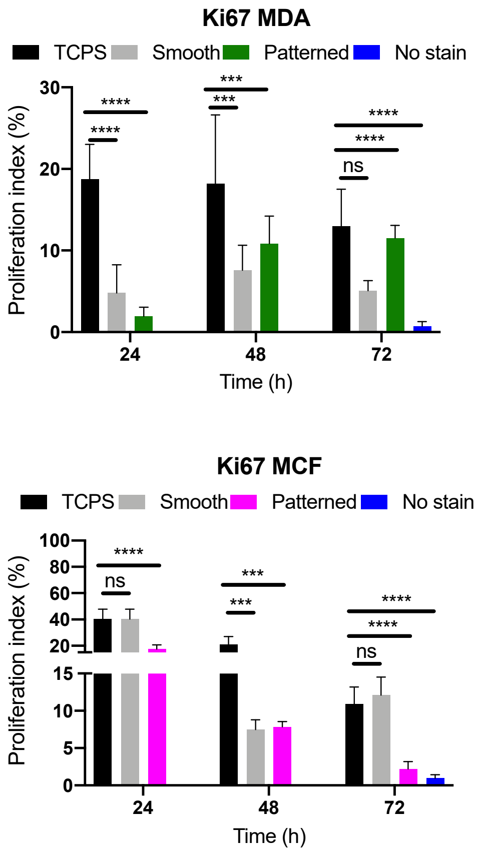

Supplementary Figure S6. Ki67 proliferation index of MCF7 and MDAMB231 cells. (Two way ANOVA, MCF7: p_{time}<0.0001, $p$sample<0.0001, MDAMB231: p_{time}=0.0468, p_{sample}<0.0001).

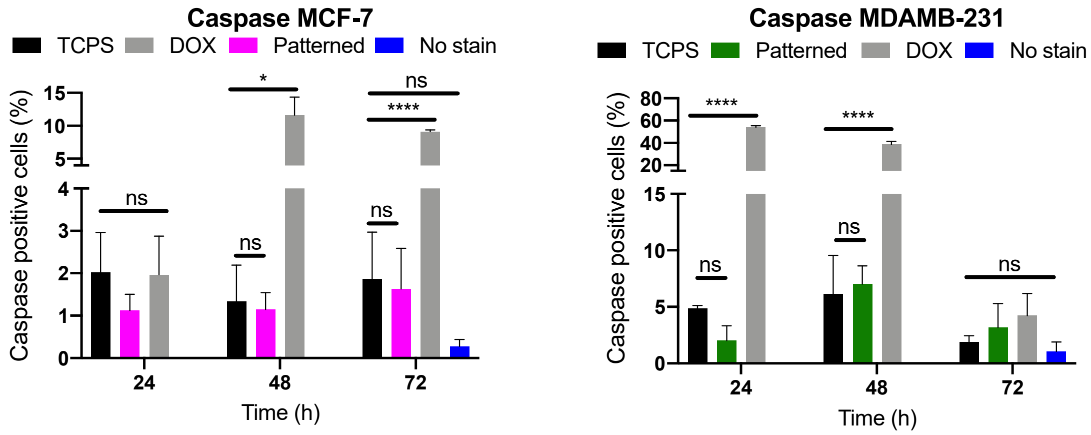

Supplementary Figure S7. Caspase analysis of MCF7 and MDAMB231 cells (Two way ANOVA, MCF7: p_{time}=0.0013, $p$sample<0.0001, MDAMB231: p_{time}<0.0001, p_{sample}<0.0001).

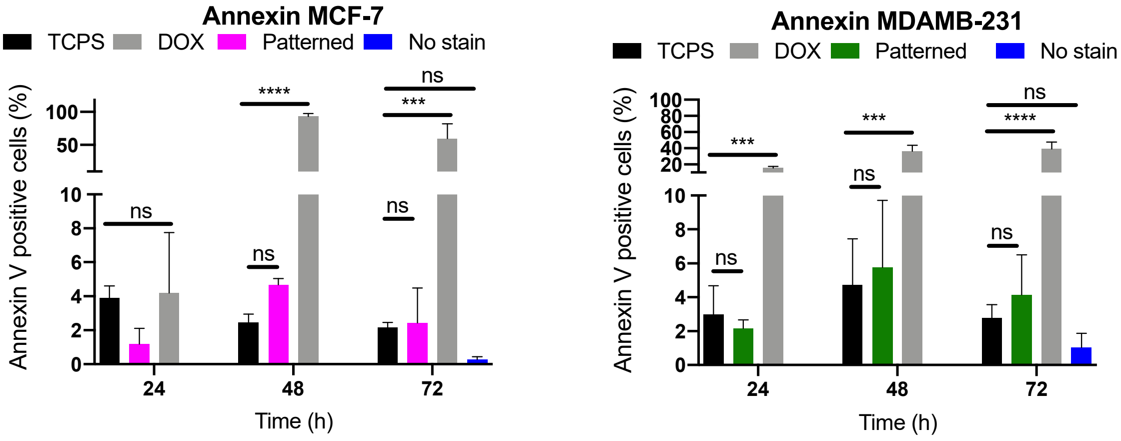

Supplementary Figure S8. Annexin V analysis of MCF7 and MDAMB231 cells (Two way ANOVA, MCF7: p_{time}=0.0013, $p$sample<0.0001, MDAMB231: p_{time}<0.0001, p_{sample}<0.0001).

Supplementary Table S1. Shape descriptors and their equations.

**Circularity**	Circularity = $4\pi \times \frac{\textbf{Area}}{[\textbf{Perimeter}]^2}$
**Feret**	Feret = $\frac{\text{Max Feret (Diameter Max)}}{\text{Min Feret (Diameter Min)}}$
**Roundness**	Roundness = $4\times \frac{\text{Area}}{\pi\times[\text{Major axis}]^2}$
**Aspect ratio**	Aspect ratio = $\frac{\text{Bounding box length}}{\text{Bounding box width}}$
**Solidity**	Solidity = $\frac{\text{Area}}{\text{Convex Area}}$

Supplementary Table S2. Primer forward and reverse sequences used for qRT-PCR experiments, their NCBI accession numbers and their references.

Gene	**NCBI Acces. No.**	**Forward**	**Reverse**	**Ref.**
GAPDH	NM_001289746.1	CACCCACTCCTCCACCTTTG	CCACCACCCTGTTGCTGTAG	55
CCNA	NM_001237.3	AGCTGCCTTTCATTTAGCACTCTAC	TTAAGACTTTCCAGGGTATATCCAGTC	57
CCNB	NM_031966.3	TATGCAGCACCTGGCTAAGA	CATGCTTCGATGTGGCATAC	58
CDKN1A	NM_078467.2	ATGTGGACCTGTCACTGTCTTG	CGTTTGGAGTGGTAGAAATCTG	56
Ki67	NM_002417.4	ATTGATCGTTCCTTCAGGTATG	TCATCAGGGTCAGAAGAGAA	59
